# Status and potential clinical value of a transthoracic evaluation of the coronary arteries

**DOI:** 10.1186/s12947-016-0048-5

**Published:** 2016-01-19

**Authors:** Fabien Labombarda, Samuela Castelnuovo, Dionysis Goularas, Cesare R. Sirtori

**Affiliations:** 1 Department of Cardiology, CHU de Caen, Avenue cote de nacre, 14000 Caen, France; 2 Center E. Grossi Paoletti, University of Milano and Dyslipidemia Center, Niguarda Hospital, Milan, Italy; 3 Department of Computer Engineering, Yeditepe University, Istanbul, Turkey

**Keywords:** Coronary artery, Intima-media thickness, Ultrasound, Harmonic imaging: coronary reserve, Statins

## Abstract

The growing need for coronary evaluation has raised interest in non-radioactive, non-invasive monitoring systems. In particular, radiation exposure during coronary investigations has been shown to be a possible cause of an enhanced risk of secondary tumors. Literature search has indicated that transthoracic echocardiography (TTE) has been widely applied to coronary arteries up to 2003, following which the lack of adequate equipment and the increased availability of invasive diagnostics, has reduced interest in this low cost, low-risk technology. The more recent availability of newer, more sensitive machines, allows evaluation of a larger number of arterial trees, including the aorta in newborns, the prenatal aortic intima-media thickness, as well as the detection of coronary artery anomalies in the adult. Improved technology for this highly operator sensitive technique may thus predict a possible evolution toward the clinical diagnostics of coronary disease and, eventually, also of the progression/regression of disease. We sought to evaluate the present status of this seldom quoted non-invasive technology.

## Introduction

A number of invasive and non-invasive methods allows, as of now, a reliable assessment of the coronary artery status. This may indicate, in turn, the area of coronary lesion/s to be addressed to invasive treatments, such as bypass surgery or percutaneous transluminal angioplasty (PTCA). A more general evaluation of the coronaries, not necessarily aimed to a possible invasive correction, is by CT scan (coronary calcium score) [1], providing a general indication on the presence of significant atheroma, or by magnetic resonance imaging (MRI) [2]. This latter is generally carried out in the context of a functional study, directed, eg to the evaluation of impending heart failure or of genetic myopathies [3].

While the strategy for coronary disease evaluation is quite well accepted, still a number of factors indicate the need for changes in this strategy. The fact that invasive coronary evaluation most frequently leads to a coronary intervention, may be criticized based on the data from the COURAGE trial [4], negating a clear benefit of surgical or angiographic correction of the detected lesion vs a conservative approach. Further, and most disturbing, the present methods for coronary evaluation are the object of criticism because of the high cost and long duration of the test, eg in the case of cardiac MRI, or, still more disturbing, because of the high risk of radiation exposure [5]. This is the case of all angiographic evaluations and, more so, of the evaluation of the calcium score by CT scans. Radiation exposure may lead to an enhanced risk of secondary tumors, in particularly breast cancer in women [6]. and lymphomas or leukemias/lymphomas after CT exposure in childhood and adolescence [7, 8].

In view of these objections on the present status of diagnostic procedures for coronary artery disease, interest has remained high on a possible non-invasive diagnosis of coronary lesions, not associated to radiation exposure. This type of technology had an initial, partially successful development in the mid-90s [9, 10], but interest faded in the early 2000 [11, 12]. The present availability of more sophisticated ultrasound equipment makes it worthy to re-examine early achievements with this technique, also in view of a possible use of therapeutic approaches requiring the direct evaluation of coronary wall changes.

The aims of this review are to outline the application of TTE for the coronary arteries analysis and the potential clinical use.

## Review

### Transthoracic echocardiography – application to the coronary arteries

Transthoracic echocardiography (TTE) is of current daily use in the diagnosis of valvular disease and of left ventricular function (ejection fraction) [13]. TTE applications have been improved, in more recent years, by the development of harmonic imaging and of ultrasound contrast agents. Even without the use of these latter, TTE may offer an attractive strategy for coronary evaluation and, potentially, to monitor drug or recombinant product therapies.

Initial application of TTE to the coronary arteries was by way of a mechanical sector scanner incorporating a dynamically focused 7.5 mHz annular phased array transducer. The system, providing a resolution of 0.2 mm, had the advantage of allowing selection of strata of the cardiac tissue, facilitating vision and selection of individual images. With this technology the Authors earlier evaluated the left anterior descending (LAD) coronary in transplanted hearts [9] both longitudinally and cross-sectionally.

The more readily visualisable LAD suggested a direct approach to this single, most important arterial tree. Using a TTE system, about 2 cm of the more proximal part of the artery can be directly visualized by high-resolution two-dimensional echo [9]. Interestingly, in this early evaluation, it was apparent that LAD wall thickness was about twice higher (1.9 vs 0.9 mm) vs thickness estimated by an intravascular ultrasound (IVUS) in a comparable series of coronary patients [11, 12]. This difference in estimated thickness led to an almost doubling of the external coronary diameter with little difference in internal diameter (with the exception of a single coronary patient with LAD occlusion) [12]. The Authors also described a higher echogenicity in the coronaries from patients vs normals [12]. This latter observation may fit with the known different mechanical properties of diseased arteries, as assessed by IVUS elastography, in particular a high strain value for fatty vs fibrous plaques, identifying macrophage-rich areas [14]. These different mechanical properties may result in altered ultrasonic properties resulting in the detection of a thickened coronary wall by TTE.

In a more detailed evaluation of LAD by TTE US [15] increased thickness in diseased arteries could be documented for the intima-media, but also for the adventitial portion of the arterial wall. The adventitia, according to the Authors, could be well represented by a third, external vascular layer, not evaluable by IVUS, and most likely responsible for the raised thickness. The identity of this layer has yet to be clarified. It might possibly explain the discrepancy between coronary wall changes measured by IVUS and by MRI. In particular, IVUS changes after statins, both at the coronary artery level, and in other arteries are generally of a minimal degree (more frequently just stabilization) [16], versus clearer wall changes (including reduced edema of the lesioned area) after MRI [17].

Advantages and drawback of IVUS, MRI and high resolution TTE for the coronary arteries investigation are summarized in Table 1.

**Table 1:** Advantages and drawback of Intra vascular ultrasound, magnetic resonance imaging and high resolution trans thoracic echoacardiogram for the coronary artery investigation

Method	Advantages	Drawbacks
Intra Vascular Ultra Sound	High spatial resolution	Invasive nature
	Wall thickness measurement	
	Three layers coronary artery	
	Characterization of the vulnerable plaque	
	Availability	
	Safety wall vizualization	
Magnetic Resonance Imaging	Wall thickness measurement	Low spatial resolution
	Characterization of the vulnerable plaque	Technically challenging (cardiac and respiratory motion, small size and non linear course of the coronary vessels)
	Magnetic resonance angiography	
	Safety	Low availabitity
		High cost
High Resolution Trans Thoracic Echocardiogram	Wall thickness measurement	No visualization of the entire coronary arteries
	High spatial resolution	No characterization of the vulnerable plaque
	Doppler information	
	Availability	Need for a good acoustic window
	Safety	Artifact if calcification
	Low cost	

**Fig. 1:**
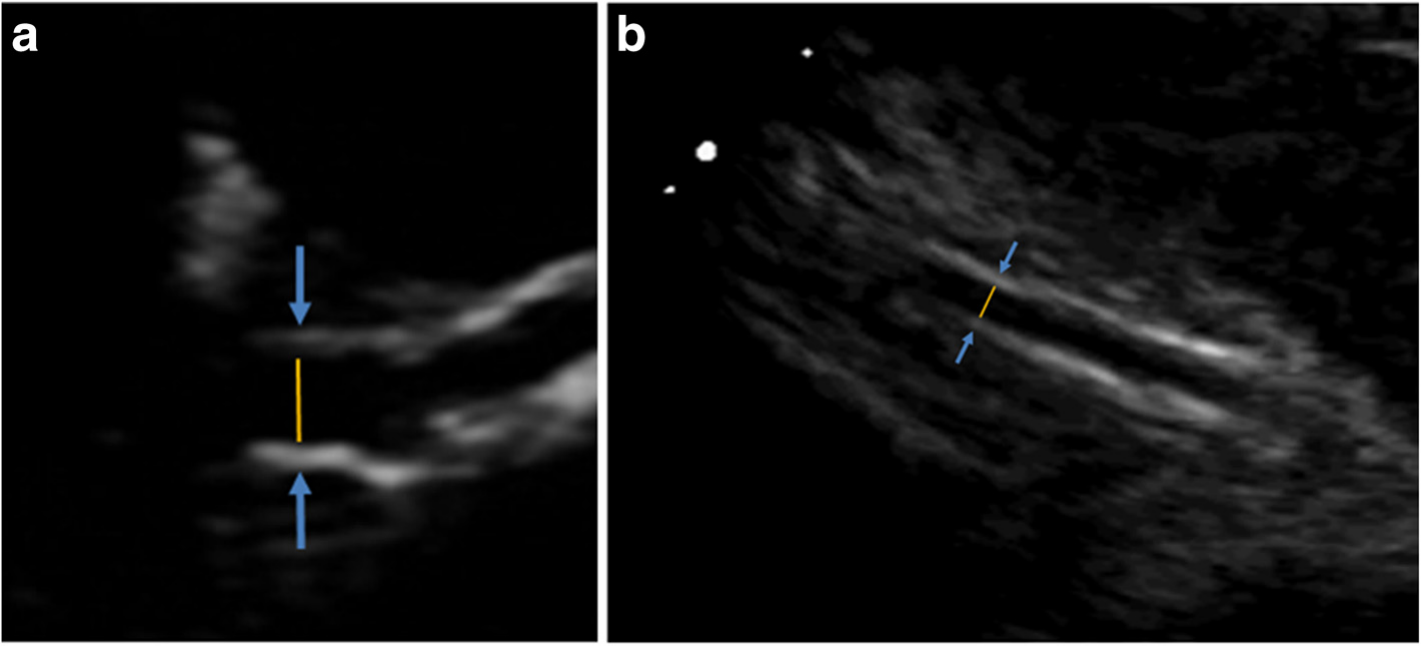
Images of Left Main Coronary Artery (Panel **a**) and Right Coronary artery (segment 2, Panel **b**) by trans thoracic echocardiography. Coronary artery walls are visible as two linear echos. Yellow line: luminal diameter of the vessel; blue arrowheads: the external boundary of the vessel

Technologies have been described also in order to achieve TTE visualization of all three coronary arteries. This has been accomplished by careful anatomical studies of the heart structure and by the use of a 5.0 or 3.5 MHz narrow-band sector transducer in a second harmonic mode for B-mode examination. The proposing Authors indicated that harmonic imaging considerably facilitates evaluation [18]. Tissue harmonic imaging (THI) is a signal processing technique in the ultrasound domain that creates images based on the differences between harmonic frequencies transmitted through tissue [19]. Since THI provides better tissue contrast and improve the signal-to-noise ratio, THI has gained popularity in cardiac evaluation. This technology is mainly used for defining tissue-of-interests (e.g. endocardial border [20], left ventricular thrombi [21]) and for echocardiographic measurement (e.g. left ventricular volume [22]), but it can be additionally utilized for coronary evaluation. The evaluation of all three coronary arteries followed established techniques. While left main and LAD, can be visualized in a parasternal axis view (Fig. 1, Panel a), the circumflex (Cx) is visualized in the parasternal short axis view, just below the left atrial appendage. Finally the proximal right coronary (RCA) can be also visualized in the parasternal short axis plane by B-mode US (Fig. 1, Panel b). A similar technology was used to assess the coronary flow reserve by Doppler TTE, as in a case of Anderson/Fabry disease [23]. By TTE Doppler the Authors also described the localization of restenosis after PTCA [19].

### Doppler vs B-mode for TTE coronary evaluation

Both B-mode and Doppler US has been described as potentially of use in TTE coronary evaluation. Doppler US was initially found of value for a functional assessment of, particularly, coronary flow reserve [20]. This early study was followed by more detailed investigations until, very recently Versundsvag et al., after localization of the three main coronaries by US, determined peak systolic flow velocities by Doppler [21]. The determination of the stenotic to prestenotic velocity ratios and color aliasing, allowed to obtain high sensitivity in the diagnosis of stenoses of the LM and LAD, not in the Cx and RCA. Coronary flow by Doppler can also, eventually, rule out or diagnose a restenosis after PTCA [19], but this appears to be limited to the LAD [22] and false diagnoses may occur [24]. By a similar, more advanced technology, however, coronary flow reserve can be adequately monitored by TTE, following dipyridamole stress echocardiography. This may add prognostic value for patients with normal or near-normal coronay arteries [25].

With more recent US equipment, Authors have described unpredicted developments in the diagnostic potential of coronary US evaluation. Perry et al. have suggested the use of the LAD wall thickness as a marker of coronary disease, also providing a normal range of values [26, 27]. The same Authors also evaluated LAD IMT after statin treatment. Follow-up of treatment did not indicate, however, a clear benefit [28].

B-Mode US of TTE visualized coronaries may hold considerable promise as an alternative/additional method for the evaluation of coronary anatomy. Experience with another vascular tree, ie the carotids, now dating back almost 30 years and developed in the institution of one of the Authors [29] has led to a very widely used diagnostic tool, almost totally replacing carotid injections of contrast media used as a pre-operative procedure. In addition, the intima-media thickness (IMT) evaluation, by a well standardized technology, has become a useful method for coronary risk assessment [30], also because of the direct correlation between IVUS determined coronary IMT and US measured carotid IMT [31].

A number of reports have been provided on the aortic wall thickness in newborns [32] and more recently, and somewhat surprising, in utero [33]. These arterial segments have diameters well below 1 cm and the described IMT are generally < 800 μm. McCloskey et al. [32] in particular, described an edge-detection software and manual caliper measurements with a 4–13 mHz linear array vascular transducer, in order to measure aortic IMT in children below 33 weeks, with excellent inter- and intra-observer reproducibility. Very recently a similar technique has been applied to the evaluation of the prenatal aortic IMT [34]: the Authors also provide an algorithm that identifies the abdominal aorta and estimates its diameter and IMT from routine ultrasonographic fetal data. This technology, aimed to diagnose newborns with intrauterine growth restriction and possibly future cardiovascular risk, may hold appeal for a similar evaluation in the coronary/cardiac area.

### Technologies for coronary visualization by B-mode TTE and potential clinical use

The objective of achieving a diagnostic procedure competitive with the invasive/radiological ones can be best reached by a B-mode US method. While a Doppler flow method can provide some knowledge on coronary dynamics, it certainly falls short of an assessment of coronary disease of value in the clinical setting. The equipment and method to be used need, however, changes versus the more established methodologies used, eg, for cardiac echo imaging and for carotid IMT evaluation.

The major problem with coronary imaging is the depth of the vessel to be reached by TTE and, additionally, its small size. Equipment needs thus to have a relatively low frequency, allowing good penetration, but this should not be at the expense of lower resolution; the lower sensitivity of the Doppler exam is less damaging, since the Doppler effect is essentially used only to confirm location of the detected coronary. Wall structure and thickness seem, thus, to be the major targets for visual evaluation of the coronaries. Interestingly, and possibly confirming data giving little value to coronary inner diameter as a risk marker for coronary events, little difference was shown in external diameter between coronaries with and without significant stenosis [35].

Coronary structure, besides thickness, has shown significant differences between patients with and without disease. This apparently stems from a difference in thickness evaluation between measurements taken from an IVUS and from a TTE [12]. There appears to be an external component in the TTE US, the adventitial layer, not seen at IVUS and possibly representing an atheroma rich vascular area [12, 15], undergoing positive remodeling an eventually resulting in increased vulnerability [36]. This has been well shown also in the intracranial arteries [37].

Another area for the clinical use of a non-invasive coronary US by TTE may be the evaluation of fractional flow reserve (FFR). As recently reported by Renker et al. [38] by using CT scan and a dedicated algorithm, one can calculate FFR without the need of coronary catheterization. Additive pronostic value of FFR evaluation was shown Rigo and Picano [24, 25] this is of special value for patients with normal or near normal ECG. Full achievement of this goal will, of course, require full length visualization of each coronary artery. A recent overview on the cardiac imaging approach to the athlete’s heart suggested that echocardiography, also with the objective of assessing anomalous coronary origins, should be tested in all competitive athletes [39]. According to these Authors, coronary origins can be visualized in >90 % of the athletes using TTE.

The increasing prevalence of coronary artery disease and the limitations of invasive assessment indicate the need for non-invasive tests. Before coronary artery disease becomes clinically manifest, as assessed by direct epicardial US imaging, more than 90 % of coronary arteries are clearly diseased, even in the presence of a normal angiographic picture [40]. Practical use of a satisfactory visualization of the proximal portion of both coronary arteries will be that of a rapid, low cost evaluation of the status of the coronary system, without the need of an immediate invasive evaluation, with inherent cost, difficult organization and radiation exposure.

**Fig. 2:**
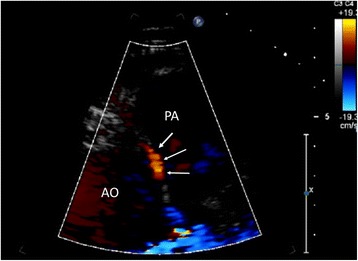
Abnormal right coronary artery from the opposite sinus with inter arterial course (arrows). Ao: aorta, PA: Pulmonary Artery

Such may be the case of congenital anomalies, as recently described by one of us [41] by 2D US examination using a 5.0 and 8.0 broadband probe (Fig. 2). More ambitiously, TTE US of the coronaries may allow assessment of wall changes after pharmacological treatments, including, eg, HDL infusions [42].

## Conclusion

TTE US of the coronaries is a potentially important diagnostic tool for coronary evaluation, both as a preliminary to an invasive procedure, or as a possible substitute. In addition, it may offer a readily accessible method for evaluating coronary changes after therapeutic procedures affecting the coronary arteries. The procedure is, at present, still operator sensitive, but the recent progress in IMT measurement in the aorta of newborns, or even in utero, with the development of appropriate software, indicates the feasibility of this approach.

## Abbreviations

CT: Computed tomography
Cx: Circumflex coronary artery
IMT: Intima-media thickness
IVUS: Intravascular ultrasound
LAD: Left anterior descending
PTCA: Percutaneous transluminal angioplasty
TTE: Transthoracic echocardiography
US: Ultrasound
